# Distinct features of three clinical subtypes in 533 patients with primary hypertrophic osteoarthropathy

**DOI:** 10.1186/s13023-025-03722-3

**Published:** 2025-04-18

**Authors:** Xilei Cai, Xiujuan Yang, Pengyue Zhang, Ziyue Dou, Zilian Chen, Chongzhi Zhu, Weiwei Xu, Xinchen Wang, Xiaodan Hong, Zhenhua Zhang

**Affiliations:** 1 Department of Infectious Diseases, The Second Affiliated Hospital of Anhui Medical University, Furong Road 678, Hefei, 230601 China; 2 Institute of Clinical Virology, The Second Affiliated Hospital of Anhui Medical University, Hefei, China

**Keywords:** Primary hypertrophic osteoarthropathy, Pachydermoperiostosis, Touraine-Solente-Gole syndrome, Clinical subtypes, Genetic subtypes

## Abstract

**Background:**

Primary hypertrophic osteoarthropathy (PHO) is a rare genetic disorder classified into clinical subtypes and genetic subtypes. Previous clinical studies have primarily focused on case reports and family analyses, largely characterizing the genetic subtypes. However, there remains a long-standing gap in understanding the characteristics of the different clinical subtypes of PHO. This study aimed to determine the distribution of the three clinical subtypes of PHO and compare their clinical characteristics using a large global sample.

**Methods:**

A systematic literature search was conducted in multiple databases to categorize cases into complete form (CO), incomplete form (IN), and fruste form (FR). Statistical analyses were performed to assess clinical differences in a retrospective study design.

**Results:**

Males predominated across all subtypes, whereas females were most prevalent in IN patients (51.1%). IN patients had the highest family history rate (62.1%). Age at onset peaked in adolescence for CO and FR patients, while IN patients exhibited bimodal peaks in early childhood and adolescence. Congenital diseases were more frequent in IN patients (7.8%, *P* = 0.021), while CO patients had a higher prevalence of digestive system diseases (12.2%, *P* = 0.007). Urinary prostaglandin E2 (PGE2) and PGE Metabolite (PGEM) were consistently elevated in CO and FR patients. In IN patients, urinary PGE2 levels were also increased, but the urinary PGEM levels showed equal proportions of elevation and reduction. Genetic analysis revealed that solute carrier organic anion transporter family member 2A1 (SLCO2A1) mutations were predominant in CO (95 cases, 73.1%) and FR (22 cases, 57.9%) patients, whereas hydroxyprostaglandin dehydrogenase (HPGD) mutations were most frequently associated with IN (25 cases, 73.5%).

**Conclusions:**

The three clinical subtypes of PHO exhibited distinct characteristics with no clear correlation between clinical and genetic subtypes. These findings highlighted the clinical significance of PHO typing and provided valuable insights for diagnosis, differential diagnosis and subtype-specific management strategies.

**Supplementary Information:**

The online version contains supplementary material available at 10.1186/s13023-025-03722-3.

## Introduction

Primary hypertrophic osteoarthropathy (PHO), also known as pachydermoperiostosis (PDP) or Touraine-Solente-Gole syndrome [1, 2], shares similar clinical features with secondary hypertrophic osteoarthropathy (SHO), including digital clubbing, pachydermia, and periostosis [3]. However, unlike SHO, which is secondary to severe cardiopulmonary diseases or malignancies [4], PHO is a rare genetic disorder caused by mutations in the hydroxyprostaglandin dehydrogenase (HPGD) gene or the solute carrier organic anion transporter family member 2A1 (SLCO2A1) gene [5, 6]. These mutations impair the transport and degradation of prostaglandin E2 (PGE2), leading to its pathological accumulation and subsequent disease manifestation [7]. PHO primarily affects the skin, bones and systemic soft tissues, particularly those involved in digital clubbing and joints abnormalities [8]. Due to its insidious onset and diagnostic challenges, many young patients experience significant functional and aesthetic impairments, impacting both psychological well-being and physiological development [9].

PHO is classified based on clinical presentation and genetic etiology. The first classification date back to the 1930s, when Touraine A, Solente G and Gole L classified PHO into three clinical subtypes based on clinical manifestations [2]: (1) Complete form (CO), with digital clubbing, periostosis and pachydermia; (2) Incomplete form (IN), presenting with digital clubbing and periostosis but minimal pachydermia; and (3) Fruste form (FR), defined by digital clubbing and pachydermia with minimal or absent skeletal involvement [10]. Genetically, PHO is classified into two primary autosomal recessive subtypes: (1) PHO autosomal recessive 1 (PHOAR1) caused by HPGD gene mutations [5]; (2) PHO autosomal recessive 2 (PHOAR2) caused by SLCO2A1 gene mutations [11]. More recently, the report of monoallelic mutations in SLCO2A1 has led to another genetic subtype, namely PHO autosomal dominant (PHOAD) [12].

Existing clinical studies on PHO are largely limited to case reports or family studies [13–16], focusing on genetic underpinnings of the disease [17–19]. These studies have identified significant differences among genetic subtypes, including sex ratio, age of onset, clinical manifestations and comorbidities. Notably, PHOAR1 typically manifests at birth, whereas PHOAR2 onset peaks during adolescence [19]. Additionally, PHOAR2 patients predominantly affects males and is frequently associated with severe anemia and gastrointestinal diseases [20, 21]. Urine PGE2 and its metabolite prostaglandin E Metabolite (PGEM) are significantly elevated in PHOAR2 compared to other genetic subtypes [22]. However, studies comparing the clinical subtypes of PHO remains scarce, with most studies limited to demographic characteristics. Although genotyping provides crucial insights into PHO pathogenesis, not all suspected patients have access to genetic testing due to regional economic and healthcare limitations. In contrast, clinical classification offers a more practical, accessible, and intuitive approach for early diagnosis and disease management. Therefore, a comprehensive retrospective analysis of the three clinical subtypes of PHO is necessary for elucidating their distinct characteristics.

This study systematically analyzed the epidemiological distribution of the three clinical subtypes of PHO and their differences in demographic characteristics, clinical manifestations, comorbidities, laboratory markers, and imaging features. Through a large-scale cohort analysis, the prevalence and distribution patterns of each subtype were quantified, and their distinct clinical characteristics were compared to assess the validity and clinical relevance of existing classification criteria. These findings enhance the precision of PHO subtype identification, improve predictions of clinical outcomes and disease progression. Furthermore, they provide critical data to support individualized treatment strategies, optimize clinical management, and inform long-term follow-up and therapeutic decision-making.

## Methods

### Search strategy and selection criteria

**Fig. 1:**
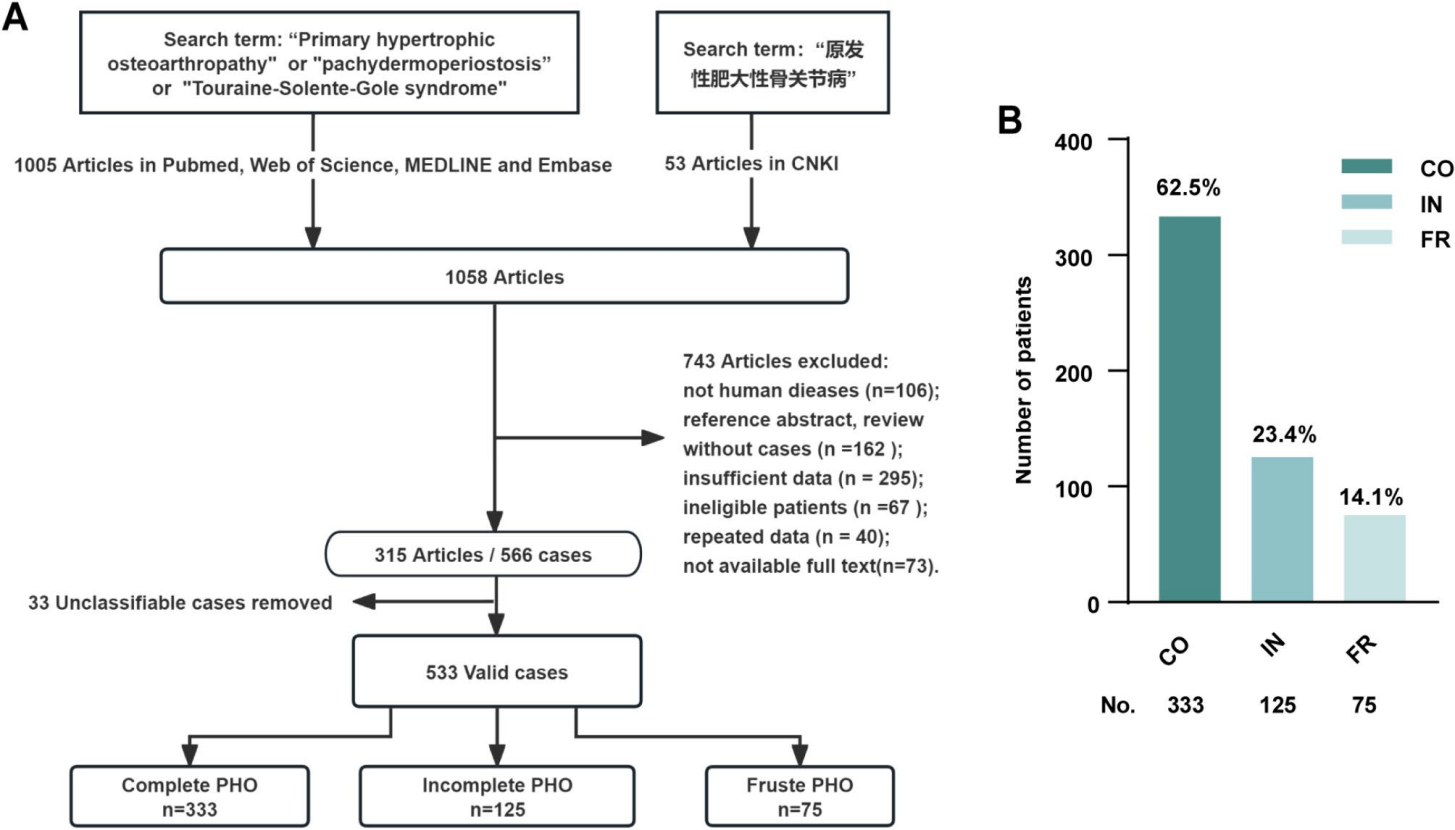
(**A**) Flow diagram illustrating the cases screening process in this study. (**B**) The number of patients categorized as CO, IN and FR across all cases. PHO: primary hypertrophic osteoarthropathy; CO: complete form; IN: incomplete form; FR: fruste form

A comprehensive literature search was conducted using the MeSH terms “Primary hypertrophic osteoarthropathy,” “pachydermoperiostosis,” and “Touraine-Solente-Gole syndrome,” along with the Chinese term “原发性肥大性骨关节病.” Databases searched included PubMed, Web of Science, MEDLINE, Embase, and CNKI. All case reports and meta-analyses related to human PHO were included, with meta-analyses traced back to the original case reports. No restrictions were applied to the publication date, but only studies published in English or Chinese were considered. Inclusion criteria for original studies were as follows: (1) Diagnosis of PHO based on digital clubbing, pachydermia, and periostosis, or confirmed by genetic testing; (2) Classification feasibility according to the criteria established by Touraine, Solente G and Gole L. Exclusion criteria were: (1) Non-case reports or non-original articles related to human PHO (e.g., reviews, commentaries, editorials); (2) Secondary hypertrophic osteoarthropathy (SHO) cases attributable to underlying pathologies (e.g., lung cancer, interstitial pulmonary fibrosis, lung abscess, tuberculosis, pulmonary lymphoma, congestive heart failure, infective endocarditis, cyanotic congenital heart disease) [23]; (3) Studies with incomplete demographic data or duplicated case records. We classified cases into subtypes using a two-step approach. First, we extracted the clinical manifestations, including digital clubbing, skin thickening, skin folds, hyperhidrosis, seborrhea, joint and bone pain, joint swelling, and effusion. Second, cases were categorized into three subtypes based on the classification system by Touraine and colleagues: complete form (CO), characterized by the presence of both periostosis and pachydermia; incomplete form (IN), defined by periostosis without pachydermia; and fruste form (FR) presenting with pachydermia and minimal or no skeletal changes [5, 11]. The Flow diagram of the cases screening process was exhibited in Fig. 1A.

### Data extraction process, data items, and quality assessment

Based on the disease characteristics of PHO and the objectives of this study, baseline information was systematically collected and categorized into the following five domains: (1) demographic data, including region, ethnicity, gender, age of onset, initial symptoms, medical history, family history, comorbidities, etc.; (2) clinical manifestations, such as digital clubbing, skin thickening, skin folds, hyperhidrosis, seborrhea, swelling and pain in joints and bones, joint effusion, etc.; (3) imaging examinations, encompassing periosteal proliferation, osteolysis, joint effusion, and other bone and joint changes, along with imaging findings of the head, chest, and abdomen; (4) laboratory indicators, such as blood routine, hormones, antibody complements, biopsies, immunohistochemistry, etc.; (5) genetic testing, covering different methods, mutation sites, amino acid changes). Data extraction and quality assessment were independently performed by two reviewers (Xilei Cai and Xiujuan Yang), with discrepancies resolved through verification and discussion with a third reviewer (Xiaodan Hong) Detailed information on all included articles was listed in Table S1.

### Statistical analysis

According to the Touraine’s classification, all cases were divided into CO, IN, and FR, respectively. Statistical analyses were conducted to compare baseline characteristics, clinical manifestations, comorbidities, laboratory indicators and genetic testing of clinical subtypes. Descriptive statistics were used to summarize the distribution of clinical classifications, with categorical variables reported as counts and percentages. Statistical analyses were performed using IBM SPSS Statistics version 27.0.1. Depending on applicability, either the Chi-square test or Fisher’s exact test was employed, with a two-sided *P* value < 0.05 considered statistically significant.

Given variations in case inclusion across time and regions, not all baseline data were consistently available. Positive and abnormal findings were more likely to be reported, while negative and unmentioned results were underrepresented. Missing information was labeled as ND (not detected). Categorical data were recorded using binary or multinomial classification. For quantitative data, we made an effort to retain the original values. To enhance the accuracy of PHO subtype characterization, two analytical approaches were employed: 1. an analysis excluding ND cases, presented in the main text; 2. an analysis including ND cases, detailed in the supplementary materials. The results from both methods were compared to ensure consistency in subtype characterization.

## Results

### Study identification and characteristics

A total of 1,058 articles related to PHO were retrieved, with 1,005 articles from foreign databases and 53 articles from Chinese databases. After applying inclusion and exclusion criteria, 315 articles were screened, including 566 cases. Subsequently, 33 cases were excluded due to the inability to classify them according to Touraine’s clinical criteria, resulting in a final dataset of 533 cases for analysis. Statistical analysis revealed that CO, IN, and FR subtypes accounted for 333 cases (62.5%), 125 cases (23.4%), and 75 cases (14.1%) of the included cases, respectively (Fig. 1B).

### Demographic characteristics analysis

#### Gender distribution

**Fig. 2:**
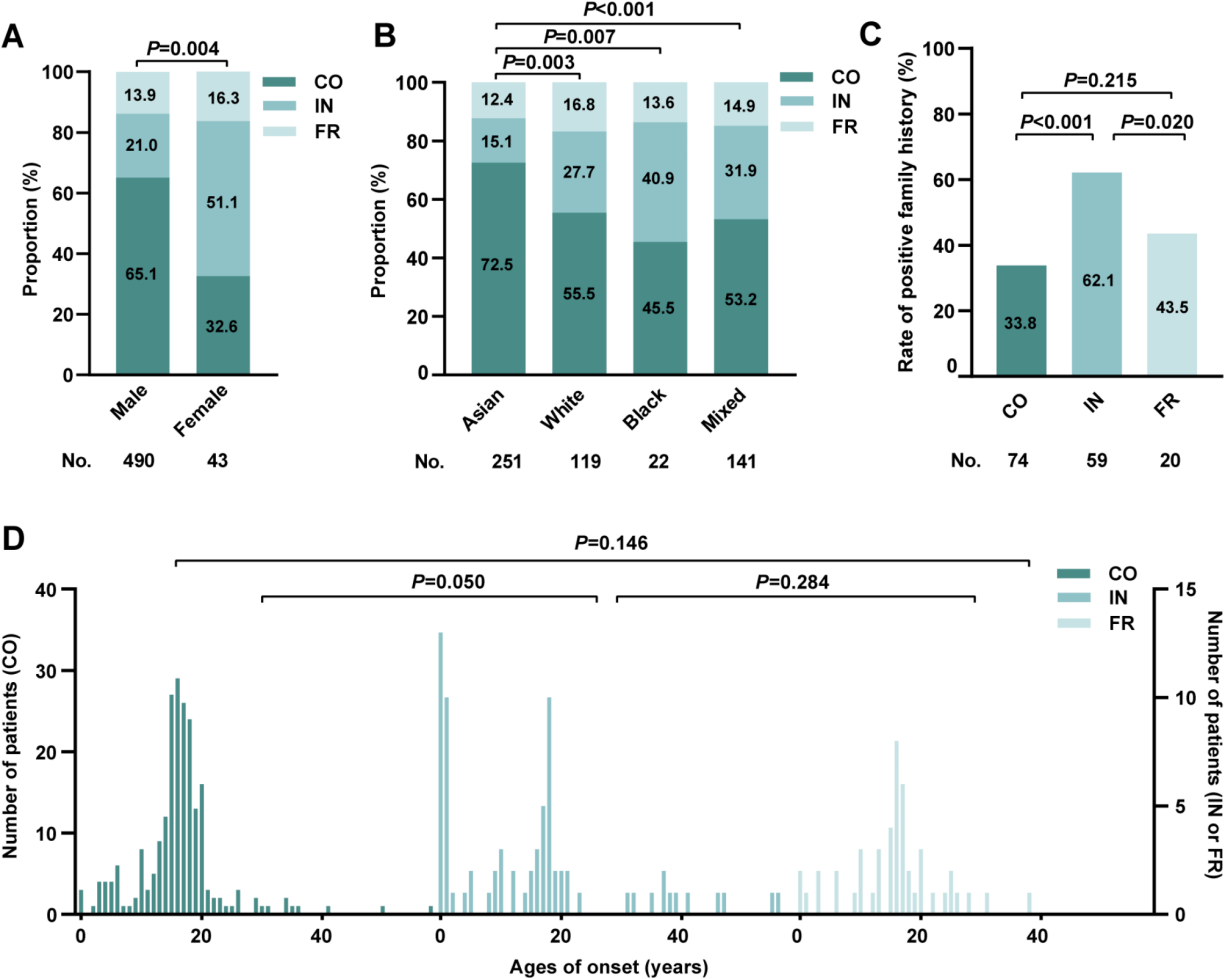
Demographic characteristics across the three clinical subtypes of PHO. Proportion of the three clinical subtypes across different gender (**A**) and races (**B**). (**C**) Rate of positive family history across each clinical subtype. (**D**) Differences in onset age across the clinical subtypes. PHO: primary hypertrophic osteoarthropathy; CO: complete form; IN: incomplete form; FR: fruste form

Our results indicated that the majority of PHO patients were male (490 cases, 91.9%), with a male-to-female ratio of approximately 11:1. This male predominance was observed across all subtypes, though the gender disparity was least pronounced in the IN subtype (male-to-female ratio ~ 5:1) (Figure S1A). Further analysis of subtype distribution by gender revealed that among male patients, CO was the common subtype (319 cases, 65.1%), with a CO: IN: FR ratio of approximately 11:3:2. In the remaining 43 female patients, the majority were IN patients (22 cases, 51.1%), followed by CO patients (14 cases, 32.6%) and FR patients (7 cases, 16.3%) (Fig. 2A). These findings indicate a significant association between gender and both PHO prevalence and subtype distribution(*P* = 0.004).

#### Racial distribution

Racial classification (Asian, White, Black, Mixed) was based on regional distribution and patient appearance to explore its association with clinical subtypes, as previous analyses rarely addressed this link. The “Mixed” category included individuals of unknown or mixed-races. In this study, Asian individuals comprised the largest proportion of PHO patients (251 cases, 47.1%) and predominated across all clinical subtypes (Figure S1B). Besides, statistical analysis revealed that the CO subtype was the most prevalent across all racial groups, with the highest proportion observed in Asian patients (182/251, 72.5%). FR patients accounted for approximately 15.0% in all racial groups, with no significant intergroup differences. A significant difference in subtype distribution was identified between Asian and non-Asian groups (all *P* < 0.05), while no significant differences were found among other racial groups (Fig. 2B).

#### Family history positive rates

Overall, 153 patients (42.5%) had a positive family history among the cases included in the study. The IN patients had the highest rate of positive family history (59 cases, 62.1%), which was significantly higher than in CO (74 cases, 33.8%, *P* < 0.001, difference: 28.3%, 95% CI: 16.7–39.9%) and FR patients (20 cases, 43.5%, *P* = 0.020, difference: 18.6%, 95% CI: 1.3–36.0%). No significant difference in rates of family positive history between the CO and FR patients (*P* = 0.215) (Fig. 2C). Findings remained consistent when ND cases were included, with the IN subtype still demonstrating the highest rate of familial occurrence (Figure S1C).

#### Age of onset

To examine age differences at onset among clinical subtypes, we defined the age of onset as the first appearance of any typical symptoms (digital clubbing, pachydermia, or periostitis). Analysis of cases with a clearly documented onset age revealed that CO and FR patients exhibited an approximately normal distribution, peaking during adolescence. In contrast, IN patients displayed a bimodal distribution, with peaks in early childhood and adolescence. A difference in onset age distribution was observed between CO and IN patients (*P* = 0.050, Fig. 2D). Due to uncertainties in onset age for some patients—particularly those seeking medical attention only after prolonged symptom presence—we further categorized patients based on an 18-year cutoff for additional analysis. The results indicated that approximately 80.0% of cases across all three subtypes had an onset age at or below 18 years (Figure S1D).

### Clinical manifestations

**Fig. 3:**
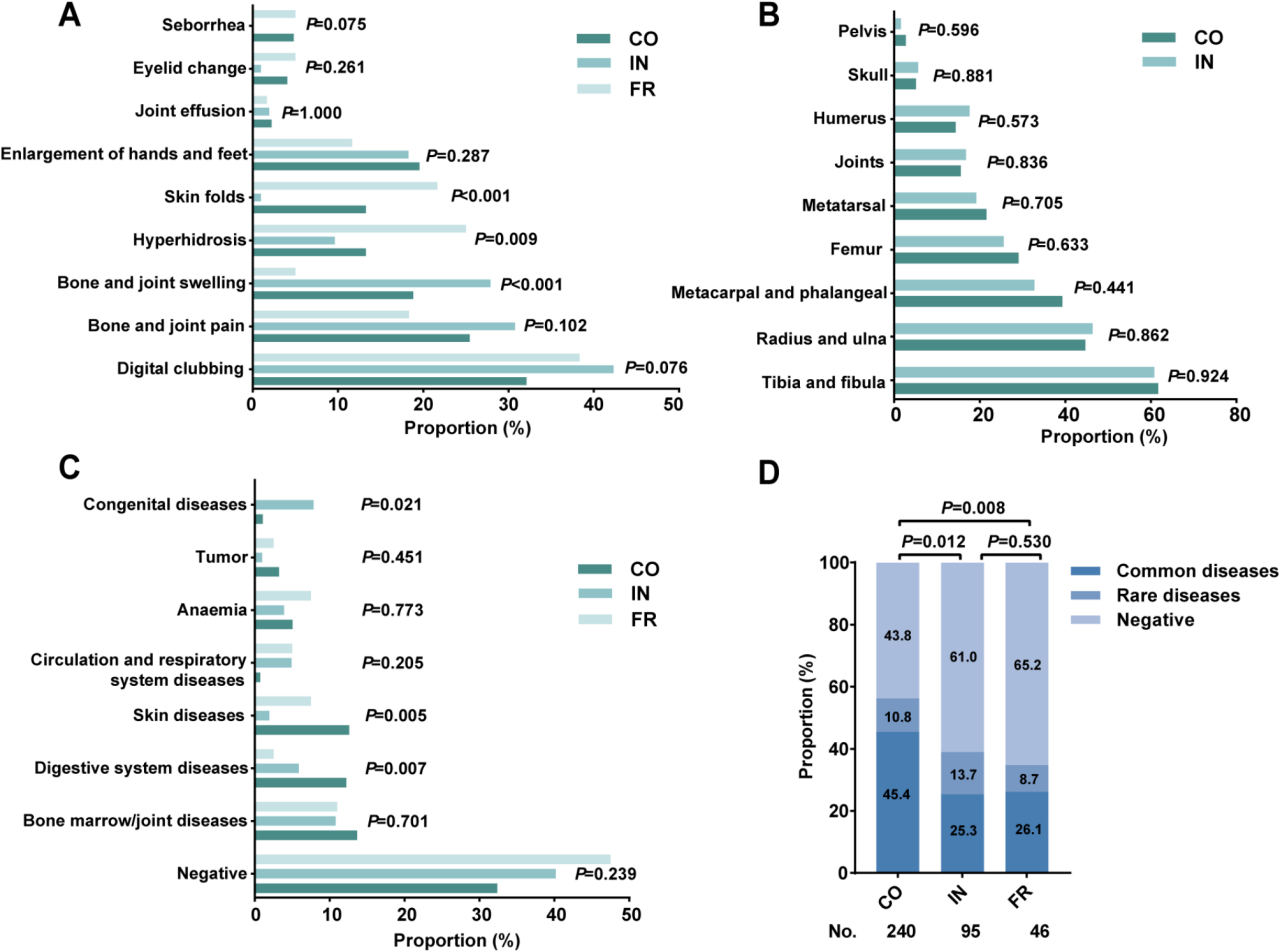
Characteristics of clinical manifestations and comorbidities across the three clinical subtypes of PHO. Differences in clinical manifestations (**A**), the site of periosteal hyperplasia (**B**) and comorbidities (**C**) across each clinical subtype. (**D**) Proportion of each type of comorbid diseases across the three clinical subtypes. PHO: primary hypertrophic osteoarthropathy; CO: complete form; IN: incomplete form; FR: fruste form

The clinical manifestations of PHO were categorized based on primary and secondary diagnostic criteria for statistical analysis [7, 10]. Digital clubbing was identified as the most prevalent clinical feature across all PHO subtypes, exceeding 30% in each subtype: 85 cases (32.1%) in CO, 45 cases (42.3%) in IN, and 24 cases (38.3%) in FR. Moreover, bones and joints swelling were significantly more frequent in IN patients (27 cases, 27.9%, *P* < 0.001), while skin folds (*P* < 0.001) and hyperhidrosis (*P* = 0.009) were most common in the FR patients (Fig. 3A). Since periosteal proliferation can affect multiple joints, such as the knee, ankle, and wrist, we compared its distribution across nine joint sites between CO and IN patients (Fig. 3B). No significant differences were detected between these two subtypes(*P* > 0.4 for all joints ). Similar trends were observed when ND patients were included in the analysis (Figure S2A).

### Comorbidities

All concurrent clinical diagnoses were collectively analyzed as PHO comorbidities. FR patients exhibited the lowest proportion of comorbidities (21 cases, 52.5%), whereas CO patients demonstrated the highest (188 cases, 67.6%), with digestive system diseases (34 cases, 12.2%) and skin diseases (35 cases, 12.6%) being significantly more prevalent than in the other subtypes (*P* < 0.05). Notably, congenital diseases were most prevalent in IN patients (7.8%, *P* = 0.021) (Fig. 3C). The inclusion of ND patients did not alter these trends (Figure S2B). Given the heterogeneity of PHO-associated diseases, comorbidities were classified into common and rare conditions. Common diseases encompassed circulatory and respiratory conditions (such as coronary heart disease, chronic obstructive pulmonary disease and emphysema), digestive system disorders (including diarrhea, ulcers, polyp and mucosal hyperplasia), as well as bone marrow/joint diseases and skin diseases (such as eczema, acne). Rare diseases included congenital conditions (such as anterior fontanelle and patent ductus arteriosus), anemia and tumors. Common diseases were predominant across all three subtypes, whereas rare diseases were significantly increased in IN patients (13 cases, 13.7%), accounting for approximately half the prevalence of common diseases (25 cases, 25.3%). The distribution of comorbidities differed significantly between CO and IN patients (*P* = 0.012) as well as between CO and FR patients (*P* = 0.008) (Fig. 3D). Upon including ND patients in the analysis, common diseases were still predominant in all three subtypes (32.7%, 19.2% and 16.0%, respectively), albeit with a noticeable decrease. Further analysis showed a significant difference in the proportion of common diseases between the CO and IN groups (difference 17.2%, 95% CI: 5.8-28.6%), and between the CO and FR groups (difference 21.4%, 95% CI: 6.5-36.3%). Similarly, the proportion of rare diseases in IN patients remained elevated but decreased to 10.4% (Figure S2C), likely due to nearly one-third of ND patients across all subtypes lacking comorbidities.

### Laboratory indicators

**Fig. 4:**
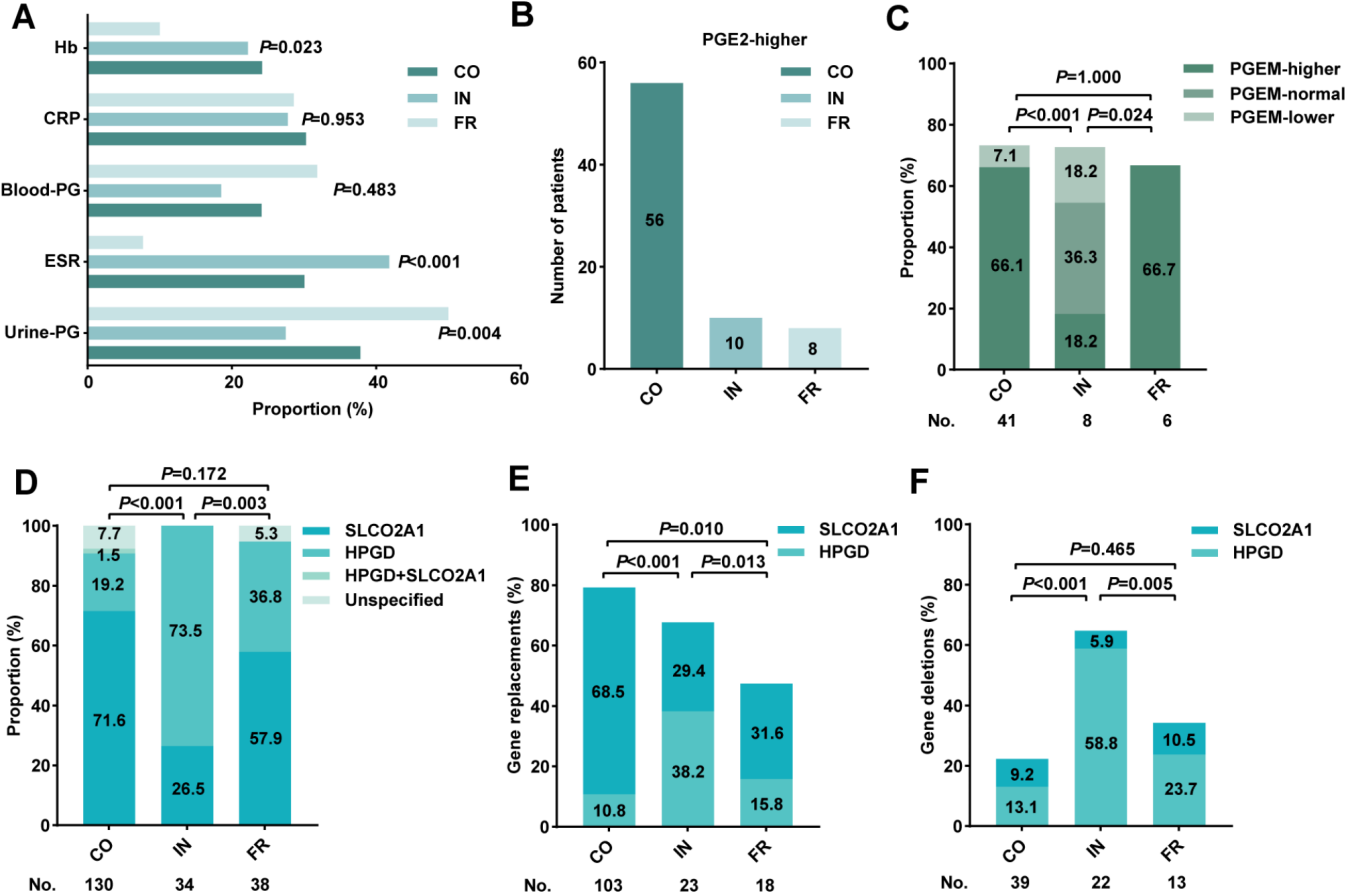
Characteristics of laboratory Indicators and genetic mutations across the three clinical subtypes of PHO. (**A**) Differences in Hb decline, ESR and CRP elevation, as well as changes in blood and urine PG levels across each clinical subtype. (**B**) The number of patients of PGE2 elevation across each clinical subtype. (**C**) Proportion of different changes in PGEM level across the three clinical subtypes. (**D**) Proportion of each type of genetic mutations across the three clinical subtypes. Comparison of gene replacements (**E**) and gene deletions (**F**) across three clinical subtypes. PHO: primary hypertrophic osteoarthropathy; Hb: hemoglobin, CRP: C-reactive protein; ESR: erythrocyte sedimentation rate; PG: prostaglandin; PGE2: prostaglandin E2; PGEM: Prostaglandin E Metabolite; CO: complete form; IN: incomplete form; FR: fruste form; SLOC2A1: solute carrier organic anion transporter family member 2A1; HPGD: hydroxyprostaglandin dehydrogenase

A statistical analysis was conducted on key laboratory indicators, including hemoglobin (Hb), C-reactive protein (CRP), erythrocyte sedimentation rate (ESR), blood and urinary hormones (mainly prostaglandins (PGs)). Our results revealed significant differences in ESR elevation (*P* < 0.001), Hb decline (*P* < 0.05), and urinary PG levels (*P* = 0.004) among the subtypes. The IN group had the highest proportion of elevated ESR (41.81%, 95% CI: 33.16–50.46%), followed by the CO group (30.06%) and the FR group (7.69%). Hb levels were significantly lower in the CO and IN groups compared to the FR group (10%, 95% CI: 3.21–16.79%). In addition, the FR group showed the highest changes in urinary PG levels (50%, 95% CI: 38.68–61.32%), exceeding those of the CO and IN groups. Urinary PG changes were detected in CO patients (62/164, 37.8%), IN patients (14/51, 27.5%), and FR patients (10/20, 50.0%), with prostaglandin E2 (PGE2) and Prostaglandin E Metabolite (PGEM) as the primary altered PG (Fig. 4A). These findings remained consistent after including ND cases (Figure S2D).

PGE2 levels were elevated across all subtypes, with 56 cases in CO, 10 in IN, and 8 in FR patients (Fig. 4B). However, PGEM alterations varied among subtypes. In the FR group, only elevated PGEM levels were observed (6 cases, 66.7%), whereas in the CO group, PGEM was elevated in 37 cases (66.1%), but decreased in 4 cases (7.1%). In contrast, PGEM alterations in the IN group were evenly distributed, with half of the affected cases showing increased levels and the other half showing decreased levels (Fig. 4C).

### Genetic mutations

HPGD and SLCO2A1 are two known pathogenic genes for PHO have been well established [5, 11]. Genetic testing data were available for CO (130/333), IN (34/125), and FR patients (38/75). Cases with unspecified genetic results were categorized separately. Statistical analysis revealed that SLCO2A1 mutations were predominated in CO patients (95/130, 73.1%), while HPGD mutations were predominated in IN patients (25/34, 73.5%). In the FR group, both gene mutations were detected, though SLCO2A1 mutations (22/38, 57.9%) were more frequent than HPGD mutations (14/38, 36.8%) (Fig. 4D). Only two CO patients exhibited co-mutations of HPGD and SLCO2A1.

To further characterize the genetic variations, gene mutations were classified as replacements, insertions and deletions. No HPGD insertions were detected. SLCO2A1 replacements were significantly more prevalent in CO patients (89/130 cases in all gene testing, 89/95 cases in SLCO2A1 mutations), compared to IN (*P* < 0.001) and FR patients (*P* = 0.010) (Fig. 4E). Conversely, partial HPGD deletions were most frequently observed in IN patients (20/34 cases in all gene testing; 20/25 cases in HPGD mutations), with significant differences compared to CO (*P* < 0.001) and FR patients (*P* = 0.005) (Fig. 4F).

## Discussion

This study systematically reviewed all available literature in English and Chinese on PHO since 1935. After removing duplicate studies and non-human studies, along with the application of rigorous inclusion and exclusion criteria, a total of 533 cases were gathered for analysis. This study marked the initial large-scale and comprehensive retrospective analysis of the distinct characteristics across the three clinical subtypes of PHO, which provided a detailed description of the features of different PHO subtypes and addressed a long-standing gap in the understanding of those. Furthermore, this study offered valuable insights for clinicians in the early diagnosis, differential diagnosis and treatment of PHO patients.

Cases were categorized into three clinical subtypes: CO, IN, and FR, based on clinical manifestations. CO was the most prevalent subtype, significantly outnumbering the other two. The key distinguishing characteristics across three clinical subtypes of PHO are summarized in Table 1. To exhibit more accurate characteristics for different PHO subtypes, we evaluated the credibility of various features by considering sample size, p-values and the differences in results between groups with and without ND patients. Ultimately, the features were classified into three categories: Highly Credible (I), Likely Credible (II), and Not Fully Confirmed (III). Category I encompassed highly credible characteristics, including a male predominance across all subtypes, a significantly higher prevalence of females in IN patients, and a markedly higher prevalence of familial history in IN patients. These findings provide robust clinical indicators for distinguishing PHO subtypes. Category II included a higher incidence of hyperhidrosis and skin folds in FR patients, a greater prevalence of congenital diseases in IN patients, and a higher tendency for digestive system diseases in CO patients. While these features are considered likely credible, additional studies are needed to confirm their diagnostic utility. Category III consisted of findings that remain inconclusive due to small sample sizes (< 50) or minor intergroup differences. These included urinary PGE2 and PGEM levels, which were typically co-elevated in CO and FR patients but showed a mixed pattern in IN patients, and Hb decline, which was most pronounced in CO and IN patients. Until further validation is available, these characteristics should be interpreted with caution in clinical practice.

**Table 1:** Comparison of the features across the three subtypes of PHO

	CO	IN	FR	Credibility
Gender	almost all males	predominantly males, with a certain proportion of females	almost all males	High
Family positive history	less prevalent	more prevalent than the other two types	less prevalent	High
Age of onset	single peak, in adolescence	double-peak, in early childhood and adolescence	single peak, in adolescence	High
Primary clinical manifestations	digital clubbing, pachydermia and periostitis	digital clubbing and periostosis, but without pachydermia	digital clubbing and pachydermia, but minimal or no skeletal changes	High
Secondary clinical manifestations				
Hyperhidrosis	less prevalent	less prevalent	more prevalent than the other two types	Likely
Skin folds	less prevalent	few	more prevalent than the other two types	Likely
Bone and joint swelling	less prevalent	more prevalent than the other two types	least prevalent	Likely
Comorbidities				
Digestive system diseases	more prevalent than the other two types	less frequent	least prevalent	Likely
Congenital diseases	much less prevalent than IN	most prevalent	not found	Likely
Urinary PGE2	elevation	elevation	elevation	Not fully confirmed
UrinaryPGEM	almost all elevation	all elevation	half elevation, half decline	Not fully confirmed
ESR elevation	less frequent than CO	most prevalent	least prevalent	Likely
Hb decline	most prevalent	most prevalent	least prevalent	Not fully confirmed
Genetic mutations	predominantly SLCO2A1	predominantly HPGD	predominantly SLCO2A1, with a certain proportion of HPGD	Likely

PHO: primary hypertrophic osteoarthropathy; CO: complete form; IN: incomplete form; FR: fruste form

Regarding comorbidities, IN patients exhibited the highest proportion of congenital diseases, including congenital malformations and birth defects [24, 25]. This finding may explain the initial peak in disease incidence after birth and the higher rate of positive family history observed in IN patients. It also highlights the need for close monitoring of congenital diseases in this patient population. However, since IN patients do not initially present with skin thickening, whether they may later progress to the CO type remains unclear and warrants by long-term follow-up.

Due to the data sources and methodological characteristics of this study, adjustment for confounding factors were not performed. Several considerations contributed to this decision. First, comorbid diseases were not comprehensively documented in the original dataset, and it was unclear whether the patients also had other conditions (e.g., osteochondral changes due to rheumatoid arthritis) that might influence the clinical presentation of PHO. Although these comorbidities could have influenced the study’s interpretation, adjusting for these confounders was not feasible. Additionally, the rarity of PHO posed another limitation. Due to its status as a rare disease, the sample size was limited, and demographic factors such as gender, age, and race were unevenly distributed across groups. This made it difficult to balance the effects of confounding factors through matching or adjustment. Lastly, the primary purpose of this study was to describe patient characteristics, clinical manifestations, and intergroup differences, rather than to explore causal relationships. As a result, descriptive statistics and simple between-group comparison methods (e.g., chi-squared test, t-test) were employed, with the interpretation of the results restricted to descriptive findings. Additionally, we compared the ND data with the results in the main text and found consistency, as detailed in the supplementary materials.

As previously mentioned, certain diseases can present with clinical features resembling PHO. Digital clubbing is observed in secondary hyperparathyroidism and acromegaly associated with scleroderma [26]. Pachydermia has been reported in various rheumatic connective tissue diseases [27]. Furthermore, periosteal changes also related to trauma or infection, Paget’s disease [28], and osteosarcoma [29]. Comorbidities refer to one or more diseases or clinical symptoms that occur independently alongside the primary disease, while complications refer to diseases or symptoms that arise during the progression of another disease, typically with a causal relationship. Given the gradual onset of PHO, determining its precise temporal relationship with other diseases is challenging. Therefore, in this study, these diseases are collectively referred to as PHO comorbidities. While their temporal relationship and potential associations require further investigation, our findings highlight the importance of addressing comorbidity treatment.

The pathogenesis of PHO involves abnormal local deposition of PGE2, which is metabolized to PGEM and excreted in urine [20]. Previous studies suggested that urinary levels of PGE2, PGEM, or their ratio can help distinguish different gene mutation subtypes of PHO [10, 22, 30]. Given that case reports primarily document altered PGE2 and PGEM levels without including unchanged results, we focused on the proportion of cases exhibiting either elevation or reduction among those tested. Our findings further support the diagnostic utility of these indicators, showing that urinary PGE2 was elevated in all three subtypes, with FR and CO patients predominantly exhibiting increased PGEM levels, while IN patients had an equal distribution of elevated and decreased PGEM levels. However, due to the limited number of cases in our study explicitly indicated increased or decreased PGE2 and PGEM levels, particularly in the IN subtype, where only two cases each showed increased or decreased urinary PGEM levels—the urinary PGE2/PGEM ratio across different clinical subtypes. Therefore, future studies with larger sample sizes are needed to validate the clinical relevance of these indicators in PHO diagnosis and classification.

Additionally, two novel biomarkers were identified in this study: a significantly higher proportion of decreased Hb and elevated ESR in CO and IN patients compared to FR patients. ESR elevation and Hb reduction are commonly associated with inflammatory and autoimmune diseases, including rheumatoid arthritis, where increased ESR and C-reactive protein (CRP) levels correlate with disease activity [31]. In addition, conditions such as infections, autoimmune diseases, chronic inflammation, and anemia may also lead to ESR elevation [32]. Since PHO presents with arthritis-like clinical symptoms, including joint pain and swelling, the observed differences in Hb and ESR levels across subtypes may reflect underlying inflammatory processes. CO and IN patients not only showed a greater prevalence of Hb reduction and ESR elevation but also more frequent joint pain and swelling compared to FR patients. Further research is required to elucidate the mechanisms underlying these hematological changes and their potential diagnostic significance in PHO subtypes. These biomarkers could potentially complement urinary PGE2 and PGEM in the diagnosis of PHO.

Our study indicated that CO and FR patients shared similar characteristics, distinct from those of IN patients. This observation potentially reflecting differences in underlying genetic mutations. Upon comparison with gene mutation subtypes, we found that CO and FR patients more closely resembled PHO patients with the SLCO2A1 mutation, whereas the characteristics of IN patients were aligned more with those of PHO patients with the HPGD mutation [5, 10, 11]. However, certain discrepancies could not be fully explained by genotype alone. For example, while PGEM levels were typically elevated in the PHO patients with SLCO2A1 mutation, some CO patients exhibited decreased levels. Additionally, severe anemia is more prevalent in the PHO with SLCO2A1 mutation but comparable in the three clinical subtypes. These variations suggest that additional genetic modifiers or mutation site variations may influence the clinical phenotype. A review of genetic testing studies suggests that both types of PHO-related gene mutations predominantly involve substitutions, with deletions occurring less frequently [20, 33–35]. Our results provided further insights, indicating that SLCO2A1 mutations were predominantly gene substitutions, whereas HPGD mutations were more frequently associated with gene deletions.

Overall, our study suggested that PHO subtypes do not strictly correspond to specific genetic mutations, highlighting the practical significance of the clinical classification approach. In clinical practice, the classification system based on clinical phenotypes remains crucial, especially in settings where genetic testing is limited or provides inconclusive results. First, the same genetic mutation (HPGD c.418G > C) may be detected in different clinical subtypes, while different mutation types may present with similar clinical features. By integrating clinical features such as prominent skin thickening and gastrointestinal symptoms in the CO subtype, laboratory findings like markedly elevated ESR in the IN subtype, and comorbidity profiles, including a higher prevalence of anemia in the FR subtype, clinicians can stratify patients into distinct subtypes to guide empirical symptomatic management. CO patients were more susceptible to digestive system diseases, potentially related to their prevalent SLCO2A1 mutations, which cause both PHO and chronic intestinal diseases [36]. Cyclooxygenase-2 (COX-2) is a key rate-limiting enzyme in PG production and PG is a crucial substance produced in the pathogenesis of PHO. Hence, COX-2 inhibitors are commonly employed in clinical practice for PHO treatment. Nevertheless, previous studies have reported that 16.7% of healthy volunteers experienced small bowel mucosal injury after using selective COX-2 inhibitors for two weeks [37]. This indicates that PHO patients, especially those with a high prevalence of gastrointestinal diseases should undergo regular blood or endoscopic monitoring during COX-2 inhibitor treatment to prevent more severe adverse effects, such as intestinal ulcers or bleeding. For patients with significant pachydermia changes, currently, only surgical treatment can directly improve their appearance. Additionally, a marked increase in ESR in IN patients often indicates underlying inflammation, which may be associated with infections, inflammatory diseases, autoimmune diseases, or certain malignancies. Therefore, enhanced observation and monitoring are necessary for early prevention and control. Meanwhile, FR patients are more prone to anemia, and further investigation and classification of the causes of anemia will provide valuable guidance for more targeted etiological treatments in the future.

Based on these findings, we propose a “clinical-genetic integrated classification” model for future diagnostic guidelines of PHO. Under this approach, empirical treatment should be initiated based on clinical presentation at the time of initial diagnosis, while genetic testing should be performed in parallel to identify potential pathogenic mutations, allowing for dynamic adjustment of therapeutic strategies. This integrated model not only improves diagnostic accuracy, but also provides a more precise framework for personalized treatment.

## Conclusions

In conclusion, our study systematically analyzed the characteristics of three PHO clinical subtypes, revealing differences in demographics, clinical manifestations, comorbidities/complications, laboratory indicators, and gene mutations. These findings highlight the clinical significance of PHO classification, bridge a long-standing knowledge gap, and provide a valuable reference for diagnosis and treatment.

## Electronic supplementary material

Below is the link to the electronic supplementary material.

Supplementary Material 1

## Data Availability

Data from public database are available on Pubmed, Web of science, MEDLINE, Embase, and the China National Knowledge Infrastructure (www.cnki.net), as reported on references and supplementary Table 1. Data can be shared upon reasonable request to the corresponding author.

## Abbreviations

PHO: Primary hypertrophic osteoarthropathy
CO: Complete form
IN: Incomplete form
FR: Fruste form
PGE2: Prostaglandin E2
PGEM: Prostaglandin E Metabolite
SLOC2A1: Solute carrier organic anion transporter family member 2A1
HPGD: Hydroxyprostaglandin dehydrogenase
PHOAR1: PHO autosomal recessive 1
PHOAR2: PHO autosomal recessive 2
PHOAD: PHO autosomal dominant
Hb: Hemoglobin
CRP: C-reactive protein
ESR: Erythrocyte sedimentation rate
PG: Prostaglandin
COX-2: Cyclooxygenase-2
