# Supplementary material for: Distinct features of three clinical subtypes in 533 patients with primary hypertrophic osteoarthropathy
Source: Orphanet J Rare Dis. 2025 Apr 18;20:188. doi: 10.1186/s13023-025-03722-3 (PMC12007382; doi:10.1186/s13023-025-03722-3)

**Supplementary information**

**Table S1.** Detailed information on all included articles in this study. CO: complete form; IN: incomplete form; FR: fruste form; SLOC2A1: solute carrier organic anion transporter family member 2A1; HPGD: hydroxyprostaglandin dehydrogenase.

**Figure S1.** Supplementary analysis of demographic characteristics across the three clinical subtypes of PHO. Gender distribution (A) and racial composition (B) across each clinical subtype. (C) Rate of positive family history acrosseach clinical subtype, including ND cases. (D) Proportion of the three clinical subtypes with onset age of 18 years or younger. PHO: primary hypertrophic osteoarthropathy; CO: complete form; IN: incomplete form; FR: fruste form; ND: not detected.

**Figure S2.** Supplementary analysis of clinical manifestations, comorbidities and laboratory Indicators across the three clinical subtypes of PHO. Differences in clinical manifestations (A) and comorbidities (B) across each clinical subtype, inclusive of ND cases. (C) Proportion of various comorbid diseases across the three clinical subtypes, including ND cases. (D) Comparison of Hb decline, ESR and CRP elevation, as well as changes in blood and urine PG levels across each clinical subtype, including ND cases. PHO: primary hypertrophic osteoarthropathy; CO: complete form; IN: incomplete form; FR: fruste form; ND: not detected; Hb: hemoglobin; ESR: erythrocyte sedimentation rate; CRP: C-reactive protein; PG: prostaglandin.

**Table S1** Detailed information on all included articles in this study

| **Author，year** | **Types of reference** | **Number of**  **total patients (n)** | **Types of**  **clinical subtype (n)** | | | **Types of**  **gene mutation (n)** | |
| --- | --- | --- | --- | --- | --- | --- | --- |
|  |  |  | **CO** | **IN** | **FR** | **HPGD** | **SLCO2A1** |
| Abdullah NRA，2017 | case report | 1 | 1 |  |  | NA | NA |
| Adams B，2016 | review | 1 | 1 |  |  | NA | NA |
| Agarwal S，2019 | case report | 1 |  |  | 1 | NA | NA |
| Akaranuchat N，2021 | case report | 1 | 1 |  |  | NA | NA |
| Akoglu H， 2009 | case report | 1 |  | 1 |  | NA | NA |
| Akrout R，2012 | case report | 1 | 1 |  |  | NA | NA |
| Alessandrella A，2018 | case study | 1 | 1 |  |  | NA | 1 |
| Alnimer Y，2017 | case report | 1 |  | 1 |  | NA | NA |
| Alves AP，2005 | case report | 1 |  |  | 1 | NA | NA |
| Anansiripun P， 2021 | case report | 1 | 1 |  |  | NA | NA |
| ANGEL JH， 1957 | case report | 3 | 3 |  |  | NA | NA |
| Arikan S，2008 | case report | 1 | 1 |  |  | NA | NA |
| Athappan G，2009 | case report | 1 | 1 |  |  | NA | NA |
| Atiase Y， 2018 | case report | 1 | 1 |  |  | NA | NA |
| Ayoub N，2015 | case study | 1 | 1 |  |  | 1 | 1 |
| Bachmeyer C， 2005 | review | 1 | 1 |  |  | NA | NA |
| Baroni A，2011 | case report | 1 | 1 |  |  | NA | NA |
| Bartolozzi G， 1975 | case report | 1 |  | 1 |  | NA | NA |
| Berdia J， 2013 | case report | 1 | 1 |  |  | NA | NA |
| Bergmann C， 2011 | case report | 5 | 3 | 1 | 1 | 4 | NA |
| Bhaskaranand K，20001 | case report | 3 | 3 |  |  | NA | NA |
| Bhate DV，1978 | case report | 3 |  | 3 |  | NA | NA |
| Bianchi L，1995 | case report | 1 |  | 1 |  | NA | NA |
| Bomanji J， 1991 | case report | 2 |  | 2 |  | NA | NA |
| Brüner S，2007 | case report | 1 | 1 |  |  | NA | NA |
| Busch J， 2012 | case report | 4 |  | 2 | 2 | NA | 4 |
| Cantatore FP，1995 | case report | 1 | 1 |  |  | NA | NA |
| Cantatore FP，1999 | case report | 2 | 2 |  |  | NA | NA |
| Castori M， 2005 | review | 1 | 1 |  |  | NA | NA |
| Chen X，2012 | review | 1 |  | 1 |  | NA | NA |
| Cheng R， 2013 | case study | 2 | 2 |  |  | NA | 1 |
| Cheng SK， 2022 | case report | 1 | 1 |  |  | NA | NA |
| Cinar F， 2006 | case report | 1 | 1 |  |  | NA | NA |
| Compton RF， 1997 | case study | 3 | 3 |  |  | NA | NA |
| Cooper RG， 1992 | case report | 2 |  | 2 |  | NA | NA |
| Cunha DJD，2020 | case report | 1 | 1 |  |  | NA | NA |
| Cunnane G， 1994 | case report | 2 |  | 2 |  | NA | NA |
| da Costa FV， 2010 | case report | 1 |  | 1 |  | NA | NA |
| Dabir T，2007 | case report | 2 |  | 2 |  | NA | NA |
| de Mestier L， 2011 | case report | 1 | 1 |  |  | NA | 1 |
| de Risi-Pugliese T，2016 | case report | 1 | 1 |  |  | NA | NA |
| de Vries N， 1986 | case report | 1 | 1 |  |  | NA | NA |
| Demirpolat G， 1999 | case report | 1 | 1 |  |  | NA | NA |
| DePadova-Elder SM， 1992 | case report | 1 | 1 |  |  | NA | NA |
| Diamond S，2007 | case report | 1 |  | 1 |  | NA | NA |
| Diggle CP， 2010 | case study | 7 | 1 | 5 | 1 | 7 | NA |
| Diggle CP，2012 | case study | 3 | 3 |  |  | NA | 3 |
| Ding J， 2013 | case report&review | 1 |  |  | 1 | NA | NA |
| Diren HB,1986 | case report&review | 6 | 1 | 4 | 1 | NA | NA |
| Dong J， 2022 | case report | 1 | 1 |  |  | 1 | NA |
| Donnelly S， 1991 | case report | 1 | 1 |  |  | NA | NA |
| Doshi D，2018 | case report | 1 | 1 |  |  | NA | NA |
| El Aoud S，2014 | case report | 1 | 1 |  |  | NA | NA |
| Erken E， 2015 | case study | 2 | 1 | 1 |  | 2 | NA |
| Fam AG，1983 | case report | 1 | 1 |  |  | NA | NA |
| Famularo G，2015 | case report | 1 |  |  | 1 | NA | NA |
| Feng Y， 2022 | case report | 1 | 1 |  |  | NA | NA |
| Fernandes GC，2011 | case report | 1 | 1 |  |  | NA | NA |
| Fietta P， 2003 | case report | 1 |  | 1 |  | NA | NA |
| Fleeter TB，1984 | case report&discussion | 1 |  |  | 1 | NA | NA |
| Fortes BC，2011 | case report | 1 | 1 |  |  | NA | NA |
| George L， 2008 | case report | 1 | 1 |  |  | NA | NA |
| Ghatnatti V， 2012 | case report | 1 | 1 |  |  | NA | NA |
| Ghosn S， 2010 | clinical trail | 3 |  |  | 3 | NA | NA |
| Giancane G， 2015 | case report&review | 4 | 2 |  | 2 | 4 | NA |
| Girisha KM，2009 | case report | 1 | 1 |  |  | NA | NA |
| González LA，2022 | edtitorial | 2 | 2 |  |  | 2 | NA |
| Gourine M，2016 | case report | 1 |  | 1 |  | 1 | NA |
| Guenter CA，1970 | case report | 1 | 1 |  |  | NA | NA |
| Guerini MB,2011 | case report | 1 | 1 |  |  | NA | NA |
| Guo T， 2017 | case report | 1 | 1 |  |  | NA | 1 |
| Guyer PB，1978 | case report | 5 | 3 | 1 | 1 | NA | NA |
| Harbison JB，1971 | case study | 4 | 3 |  | 1 | NA | NA |
| Harifi G, 2007 | case report | 1 | 1 |  |  | NA | NA |
| Harifi G, 2011 | case report | 5 | 1 | 3 | 1 | NA | NA |
| Herbert DA， 1981 | case report | 1 | 1 |  |  | NA | NA |
| Herman MA，1965 | case report | 5 | 4 |  | 1 | NA | NA |
| Hong C， 2017 | case report | 1 |  | 1 |  | NA | NA |
| Honório MLP，2020 | case report | 1 | 1 |  |  | NA | NA |
| Huang H，2017 | case study | 8 | 7 |  | 1 | 1 | 7 |
| Hussain W， 2010 | case report | 1 |  |  | 1 | NA | NA |
| Ibba S， 2016 | case report | 1 | 1 |  |  | NA | NA |
| Ikeda F，2004 | case report | 1 | 1 |  |  | NA | NA |
| Ikeda K， 2022 | case report | 3 | 2 | 1 |  | NA | 1 |
| Ishizuka T，2021 | case report | 1 | 1 |  |  | NA | 1 |
| J.C. Santos-Durán,2007 | case report | 1 | 1 |  |  | NA | NA |
| Jeyabaladevan S，2021 | case report | 1 | 1 |  |  | NA | 1 |
| Jiang Y，2019 | case report | 1 | 1 |  |  | NA | 1 |
| Jojima H，2007 | case report | 1 |  | 1 |  | NA | NA |
| Joshi A，2019 | case report | 1 | 1 |  |  | NA | NA |
| Ka MM，2002 | case report | 1 |  | 1 |  | NA | NA |
| Kabashima K，2010 | case report | 2 |  |  | 2 | NA | NA |
| Kabi F，2006 | case report | 2 | 2 |  |  | NA | NA |
| Karimova MM， 2017 | case report | 1 |  |  | 1 | NA | NA |
| Karkucak M，2007 | case report | 1 |  |  | 1 | NA | NA |
| Karnan S，2012 | case report | 1 |  | 1 |  | NA | NA |
| Kartal Baykan E， 2021 | case report | 2 | 1 |  | 1 | NA | 2 |
| Khalil G， 2022 | case report | 1 | 1 |  |  | NA | NA |
| Khan AK， 2018 | case study | 4 | 1 |  | 3 | 2 | NA |
| Kharbanda R，2020 | case report | 1 |  | 1 |  | NA | NA |
| Kim HJ，2015 | case report | 1 | 1 |  |  | NA | 1 |
| Kozlowski K，1983 | case report&review | 1 |  | 1 |  | NA | NA |
| Kudligi C， 2010 | case report | 1 |  | 1 |  |  |  |
| Kumar S，2013 | case report&review | 1 | 1 |  |  | NA | NA |
| Kumar U， 2008 | case report | 1 | 1 |  |  | NA | NA |
| Lam SK， 1983 | case report | 3 | 2 |  | 1 | NA | NA |
| Latos-Bielenska A， 2007 | case report | 5 | 2 | 1 | 2 | NA | NA |
| Lazarus JH， 1973 | case report | 1 | 1 |  |  | NA | NA |
| Lee S， 2016 | case report | 6 | 6 |  |  | NA | 6 |
| Lee SC， 1998 | case report | 1 |  |  | 1 | NA | NA |
| Leibowitz MR， 1983 | case report | 1 |  |  | 1 | NA | NA |
| Levin SE， 2002 | case report | 2 |  | 2 |  | NA | NA |
| Li N， 2020 | case study | 1 | 1 |  |  | NA | 1 |
| Li S，2015 | case report&review | 1 | 1 |  |  | NA | NA |
| Li SS，2017 | clinical trail | 43 | 35 | 8 |  | 6 | 37 |
| Li Z， 2018 | case report | 1 |  |  | 1 | NA | 1 |
| Limenis E， 2021 | case report | 1 |  | 1 |  | 1 | 0 |
| Lin MH，2018 | case report | 1 |  | 1 |  | NA | NA |
| Liu CY， 2014 | case report | 1 |  |  | 1 | NA | NA |
| Long B， 2022 | case report | 1 |  | 1 |  | NA | 1 |
| Loredo R，1996 | case report | 1 |  | 1 |  | NA | NA |
| Madruga Dias JA，2014 | case report&review | 1 | 1 |  |  | NA | 1 |
| Mahesh M， 2013 | case report&review | 1 | 1 |  |  | NA | NA |
| Mangupli R，2017 | case report | 1 | 1 |  |  | NA | NA |
| Marie I， 1999 | case report | 1 |  | 1 |  | NA | NA |
| Marques P，2020 | case report | 1 |  |  | 1 | 1 | NA |
| Martínez-Lavín M， 1993 | case report | 4 |  | 4 |  | 4 | NA |
| Martínez-Lavín M，1988 | case report | 5 |  | 3 | 2 | NA | NA |
| Matucci-Cerinic M，1988 | case report | 1 | 1 |  |  | NA | NA |
| Matucci-Cerinic M，1989 | case report | 1 | 1 |  |  | NA | NA |
| MCCORD MC，1952 | case report | 1 |  | 1 |  |  |  |
| Metz EN，1965 | case report | 4 | 2 | 1 | 1 | NA | NA |
| Minakawa S，2016 | case report | 1 | 1 |  |  | NA | 1 |
| Mishra GK，1988 | case report&review | 1 | 1 |  |  | NA | NA |
| Mittal A， 2019 | case report | 1 | 1 |  |  | NA | NA |
| Mobini M， 2018 | case report&review | 1 | 1 |  |  | NA | NA |
| Monteiro E， 2003 | case report | 1 |  |  | 1 | NA | NA |
| Mukherjee B， 2016 | case report | 1 |  |  | 1 | NA | NA |
| Nakahigashi K， 2013 | case report | 1 | 1 |  |  | 0 | 0 |
| Nakajima M， 2008 | case report | 1 | 1 |  |  | NA | NA |
| Nakazawa S， 2015 | case report | 1 | 1 |  |  | 1 | NA |
| Nakazawa S， 2017 | case report | 1 | 1 |  |  | NA | 1 |
| Nakazawa S，2018 | case report | 1 |  |  | 1 | NA | NA |
| Narayanan S， 2010 | case report | 2 | 2 |  |  | NA | NA |
| Nayak HK，2012 | case report&review | 1 |  | 1 |  | NA | NA |
| Neiman HL，1974 | case report | 1 | 1 |  |  | NA | NA |
| Ninomiya S， 2011 | case report | 1 | 1 |  |  | NA | NA |
| Ohata K，2009 | case report&review | 1 | 1 |  |  | NA | NA |
| Oikarinen A， 1994 | case report | 3 | 3 |  |  | NA | NA |
| Oiwa T， 2021 | case study | 4 |  |  | 4 | NA | 4 |
| Okten A，2007 | case report | 2 |  | 2 |  | NA | NA |
| Ozdemir M， 2007 | case report | 1 | 1 |  |  | NA | NA |
| Petrig C，2003 | case report | 1 |  | 1 |  | NA | NA |
| Poormoghim H， 2012 | case report | 2 | 2 |  |  | NA | 2 |
| Prasad A，2020 | case report | 1 |  | 1 |  | NA | NA |
| Prerna， 2018 | case report | 1 | 1 |  |  | NA | NA |
| Purohit M， 1980 | case report | 1 |  | 1 |  | NA | NA |
| Pushpa G， 2012 | case report | 1 | 1 |  |  | NA | NA |
| Radhakrishnan P， 2020 | case report | 3 | 1 | 2 |  | 3 | NA |
| Rahaman SH， 2016 | case report | 1 |  | 1 |  | NA | NA |
| Rajan TM，2013 | case report | 1 | 1 |  |  | NA | NA |
| Rastogi R，2009 | case report | 1 | 1 |  |  | NA | NA |
| Reginato AJ， 1982 | case report | 3 |  | 3 |  | NA | NA |
| Ren Y，2013 | case report | 1 | 1 |  |  | NA | NA |
| Rendina D， 2008 | case report | 1 |  | 1 |  | NA | NA |
| Rezgui-Marhoul L，2005 | case report | 2 | 1 | 1 |  | 0 | 0 |
| RIMOIN DL. 1965 | case report&review | 2 | 2 |  |  | NA | NA |
| Rostom H， 2019 | case report | 1 | 1 |  |  | NA | NA |
| Rostom H，2019 | case report | 1 |  | 1 |  | 1 | NA |
| Ryu MR， 2019 | case report | 1 |  |  | 1 | 1 | NA |
| Saadeh D，2014 | case study | 3 | 1 |  | 2 | NA | 2 |
| Salah BI， 2019 | case report&review | 1 | 1 |  |  | NA | NA |
| Samaranayake M，2010 | case report | 1 |  | 1 |  | NA | NA |
| Sandoval AR，2013 | case report | 1 | 1 |  |  | NA | NA |
| Santhosh S，2011 | case report | 1 |  | 1 |  | NA | NA |
| Sarkar RN,1999 | case report | 1 | 1 |  |  | NA | NA |
| Sasaki T， 2012 | case report | 6 | 5 |  | 1 | NA | 4 |
| Savin JA. 1968 | case report | 1 | 1 |  |  |  |  |
| Secchin P， 2019 | case report | 1 | 1 |  |  | NA | NA |
| Seggewiss R， 2003 | case report | 1 |  | 1 |  | NA | NA |
| Seifert W，2009 | case report | 2 |  | 2 |  | 2 | NA |
| Seifert W，2012 | case report | 4 | 2 |  | 2 | NA | 4 |
| Seta V，2017 | case report | 2 | 1 |  | 1 | 1 | 1 |
| Sethuraman G， 2006 | case report | 1 | 1 |  |  | NA | NA |
| Sharma ML,,2014 | case report | 1 | 1 |  |  | NA | NA |
| SHAWARBY K， 1962 | case report&review | 4 | 3 | 1 |  | NA | NA |
| Shim YW，1997 | case report | 1 | 1 |  |  | NA | NA |
| Shimizu C， 1999 | case report | 1 | 1 |  |  | NA | NA |
| Shin KC， 2009 | case report | 1 | 1 |  |  | NA | NA |
| Shinjo SK， 2007 | case report | 1 |  | 1 |  | NA | 0 |
| Siddiqui MR， 2012 | case report | 1 | 1 |  |  | NA | NA |
| Silva Dda F，2008 | case report | 1 | 1 |  |  | NA | NA |
| Singh GR， 1995 | case report | 1 | 1 |  |  | NA | NA |
| Sirinavin C， 1982 | case report | 1 | 1 |  |  | NA | NA |
| Sivathapandi T， 2018 | case report | 1 |  | 1 |  | NA | NA |
| Souto Filho JTD,2020 | case report | 1 |  |  | 1 | NA | NA |
| Stephan C，2018 | case study | 1 |  |  | 1 | NA | NA |
| Stoker DJ，1992 | case report | 1 | 1 |  |  | NA | NA |
| Sun F，2018 | case report | 1 | 1 |  |  | NA | 1 |
| Sun K， 2021 | case report | 1 | 1 |  |  | NA | 1 |
| Sun XF， 2011 | case report | 2 | 2 |  |  | NA | NA |
| Supradeeptha C，2014 | case report&review | 1 | 1 |  |  | NA | NA |
| Susmano A，1967 | case report | 1 |  | 1 |  | NA | NA |
| Synder MA， 2019 | case report | 1 | 1 |  |  | NA | NA |
| Tabatabaei SA， 2019 | case report | 1 | 1 |  |  | 0 | 0 |
| Tanaka H， 1991 | case report | 2 |  | 2 |  | NA | NA |
| Tanese K， 2015 | case report | 6 |  |  | 6 | NA | 5 |
| Tay YK，1993 | case report&review | 1 | 1 |  |  | NA | NA |
| Thappa DM，2000 | case report | 1 | 1 |  |  | NA | NA |
| Thomas RH，1985 | case report | 1 | 1 |  |  | NA | NA |
| Tinoco-Fragoso F，2015 | case report | 1 | 1 |  |  | NA | NA |
| Toepfer M,2002 | case report | 1 | 1 |  |  | NA | NA |
| Torgutalp M，2019 | case report | 1 | 1 |  |  | 0 | 1 |
| TORNBLOM N， 1959 | case report | 5 | 2 | 3 |  | NA | NA |
| Tüysüz B，2014 | case report | 1 |  |  | 1 |  |  |
| Ukinc K，2007 | case report | 1 | 1 |  |  | NA | NA |
| Ursing B.1970 | case report | 1 |  |  | 1 | NA | NA |
| Vaidya B， 2019 | case report | 2 | 2 |  |  | 0 | 0 |
| Venencie PY，1988 | case report | 1 | 1 |  |  | NA | NA |
| Villarreal-Martínez A， 2018 | case report | 2 | 2 |  |  | 1 | 2 |
| Viola IC,2000 | case report | 3 |  | 3 |  | NA | NA |
| VOGL A， 1962 | case report | 2 |  | 1 | 1 | NA | NA |
| Wagner F， 1987 | case report | 1 | 1 |  |  | NA | NA |
| Warwas S，2013 | case report | 1 |  | 1 |  | NA | NA |
| Waszczykowski M，2013 | case report | 1 | 1 |  |  | NA | NA |
| Willis E， 2020 | case report | 1 |  | 1 |  | NA | NA |
| Wright-Pascoe RA，2003 | case report | 1 | 1 |  |  | NA | NA |
| Xu C， 2021 | case report | 1 | 1 |  |  | NA | 1 |
| Younes M， 2005 | case report | 1 | 1 |  |  | NA | NA |
| Yousaf M，2022 | case report | 3 | 3 |  |  | NA | NA |
| Yuan L， 2018 | case report | 5 | 5 |  |  | NA | 5 |
| Yuan L，2015 | case study | 9 | 7 | 1 | 1 | 9 | NA |
| Yüksel-Konuk B， 2009 | case study | 3 | 2 | 1 |  | 3 | NA |
| Zhang Q，2013 | case report | 1 | 1 |  |  | NA | NA |
| Zhang Z， 2012 | case study | 3 | 3 |  |  | NA | 3 |
| Zhang Z， 2014 | case report | 1 | 1 |  |  | NA | 1 |
| Zhang Z，2013(1) | case study | 1 | 1 |  |  | NA | 1 |
| Zhang Z，2013(2) | case study | 7 | 2 |  | 5 | NA | 7 |
| 边焱焱，2012 | case report | 1 | 1 |  |  | NA | NA |
| 常成荣，2004 | case report | 1 | 1 |  |  | NA | NA |
| 陈超，2011 | case report | 1 | 1 |  |  | NA | NA |
| 陈丽华，2003 | case report | 1 |  | 1 |  | NA | NA |
| 陈瑛，2002 | case report&review | 1 | 1 |  |  | NA | NA |
| 陈志慧，2010 | case report | 1 | 1 |  |  | NA | NA |
| 崔冉，2018 | case report | 1 | 1 |  |  | 1 | 0 |
| 邓爱民，2009 | case report | 1 | 1 |  |  | NA | NA |
| 邓静敏，2012 | case report | 1 | 1 |  |  | NA | NA |
| 丁晨召，2016 | case report | 1 | 1 |  |  | NA | NA |
| 董颖越，2015 | case report | 1 |  |  | 1 | NA | NA |
| 都雪朝，2015 | case report | 1 | 1 |  |  | NA | NA |
| 方杰，2012 | case report | 1 | 1 |  |  | NA | NA |
| 甘戈，2005 | case report | 1 | 1 |  |  | NA | NA |
| 侯勇，2006 | case report&review | 5 | 3 | 2 |  | NA | NA |
| 胡婕，2011 | case report | 1 | 1 |  |  | NA | NA |
| 胡志，2005 | case report | 1 |  | 1 |  | NA | NA |
| 黄慧，2009 | case report | 1 | 1 |  |  | NA | NA |
| 贾海燕，2010 | case report | 1 | 1 |  |  | NA | NA |
| 焦彬，2014 | case report | 1 | 1 |  |  | NA | NA |
| 金萍，2018 | case report | 2 | 2 |  |  | NA | 2 |
| 李安敏，2006 | case report&review | 1 | 1 |  |  | NA | NA |
| 李德强，2006 | case report | 2 |  | 2 |  | NA | NA |
| 李东明，2014 | case report | 1 | 1 |  |  | NA | NA |
| 李丽，2013 | case report | 1 | 1 |  |  | NA | NA |
| 李青，2008 | case report | 2 | 2 |  |  | NA | NA |
| 李彤寰，2008 | case report&review | 1 | 1 |  |  | NA | NA |
| 李彤寰，2012 | case report | 1 | 1 |  |  | NA | NA |
| 李岩，2016 | case report | 1 | 1 |  |  | NA | NA |
| 李彦希，2011 | case report | 1 | 1 |  |  | NA | NA |
| 梁乙安，2006 | case report | 1 | 1 |  |  | NA | NA |
| 刘坚，2011 | case report&review | 3 | 2 | 1 |  | NA | NA |
| 刘霜，2012 | case report | 1 |  | 1 |  | 1 | 0 |
| 刘卫红，2013 | case report&review | 1 |  | 1 |  | NA | NA |
| 刘文阁，2003 | case report | 1 | 1 |  |  | NA | NA |
| 刘晓钢，2017 | case study | 1 |  | 1 |  | 1 | 0 |
| 刘彦婷，2012 | case report | 1 |  |  | 1 | NA | NA |
| 刘永信，2008 | case report | 1 | 1 |  |  | NA | NA |
| 刘志刚，2006 | case report | 1 |  |  | 1 | NA | NA |
| 娄安妮，2009 | case report | 1 | 1 |  |  | NA | NA |
| 罗斌，2000 | case report | 1 | 1 |  |  | NA | NA |
| 吕世娟，1994 | case report | 1 | 1 |  |  | NA | NA |
| 玛依努尔，2008 | case report | 1 | 1 |  |  | NA | NA |
| 满斯亮，2009 | case report&review | 5 | 2 | 3 |  | NA | NA |
| 秦娟，2014 | case report | 1 | 1 |  |  | 0 | 1 |
| 邱敏蕾，2009 | case report | 1 | 1 |  |  | NA | NA |
| 孙志杰，2012 | case report | 4 | 4 |  |  | NA | NA |
| 田宜肥，2014 | case report | 1 | 1 |  |  | NA | NA |
| 汪群，2001 | case report | 1 | 1 |  |  | NA | NA |
| 王国锋，2006 | case report&review | 1 |  | 1 |  | NA | NA |
| 王海燕，2009 | case report | 1 | 1 |  |  | NA | NA |
| 王娇，2018 | case report | 1 | 1 |  |  | 0 | 1 |
| 王磊，2015 | case report | 1 |  | 1 |  | 1 | NA |
| 王霞，2018 | case report | 1 |  | 1 |  | 0 | 1 |
| 王燕，2012 | case report | 1 | 1 |  |  | NA | NA |
| 王颖芳，2012 | case report&review | 1 | 1 |  |  | NA | NA |
| 王震英，2012 | case report | 1 | 1 |  |  | NA | NA |
| 王智欧，2014 | case report | 1 | 1 |  |  | NA | NA |
| 文振华，2009 | case report | 1 | 1 |  |  | NA | NA |
| 吴超，2014 | case report | 1 | 1 |  |  | NA | NA |
| 向鹏月，2012 | case report | 1 | 1 |  |  | NA | NA |
| 杨皓瑜，2015 | case report | 1 | 1 |  |  | NA | NA |
| 杨雪丽，2008 | case report | 1 | 1 |  |  | NA | NA |
| 杨怡，2010 | case report | 1 | 1 |  |  | NA | NA |
| 于世荣，2010 | case report | 1 | 1 |  |  | NA | NA |
| 岳学苹，2007 | case report | 1 | 1 |  |  | NA | NA |
| 张国民，2002 | case report | 1 | 1 |  |  | NA | NA |
| 张力文，2011 | case report | 1 | 1 |  |  | NA | NA |
| 张璐璐，2022 | case report | 1 | 1 |  |  | NA | NA |
| 张添，2012 | case report | 2 | 1 |  | 1 | NA | NA |
| 张巍，2002 | case report | 1 |  | 1 |  | NA | NA |
| 张云凤，2012 | case report | 1 | 1 |  |  | NA | NA |
| 周南，2009 | case report | 1 |  | 1 |  | NA | NA |
| 邹清旭，2001 | case report | 1 | 1 |  |  | NA | NA |

**Figure S1**


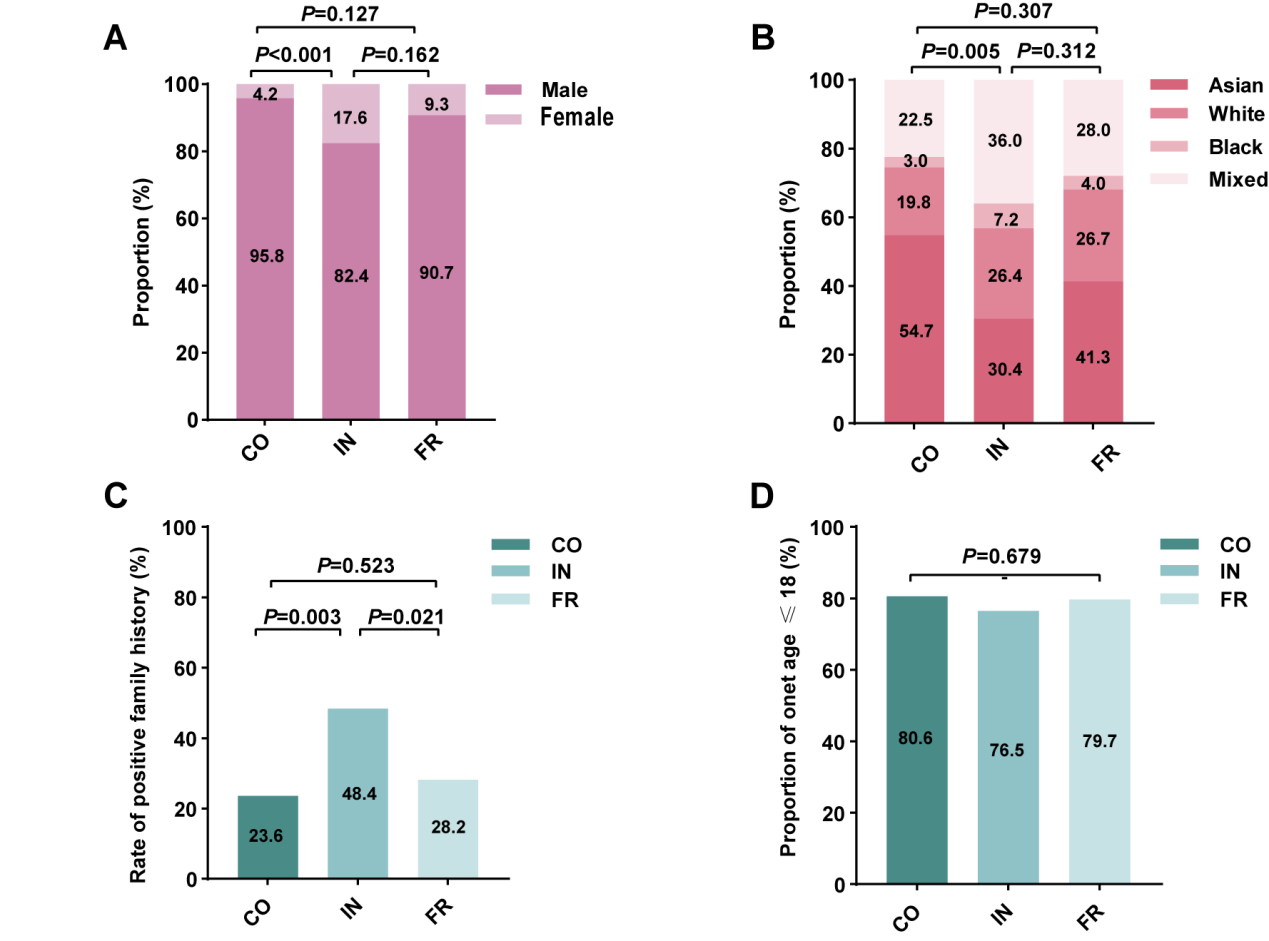


**Figure S2**


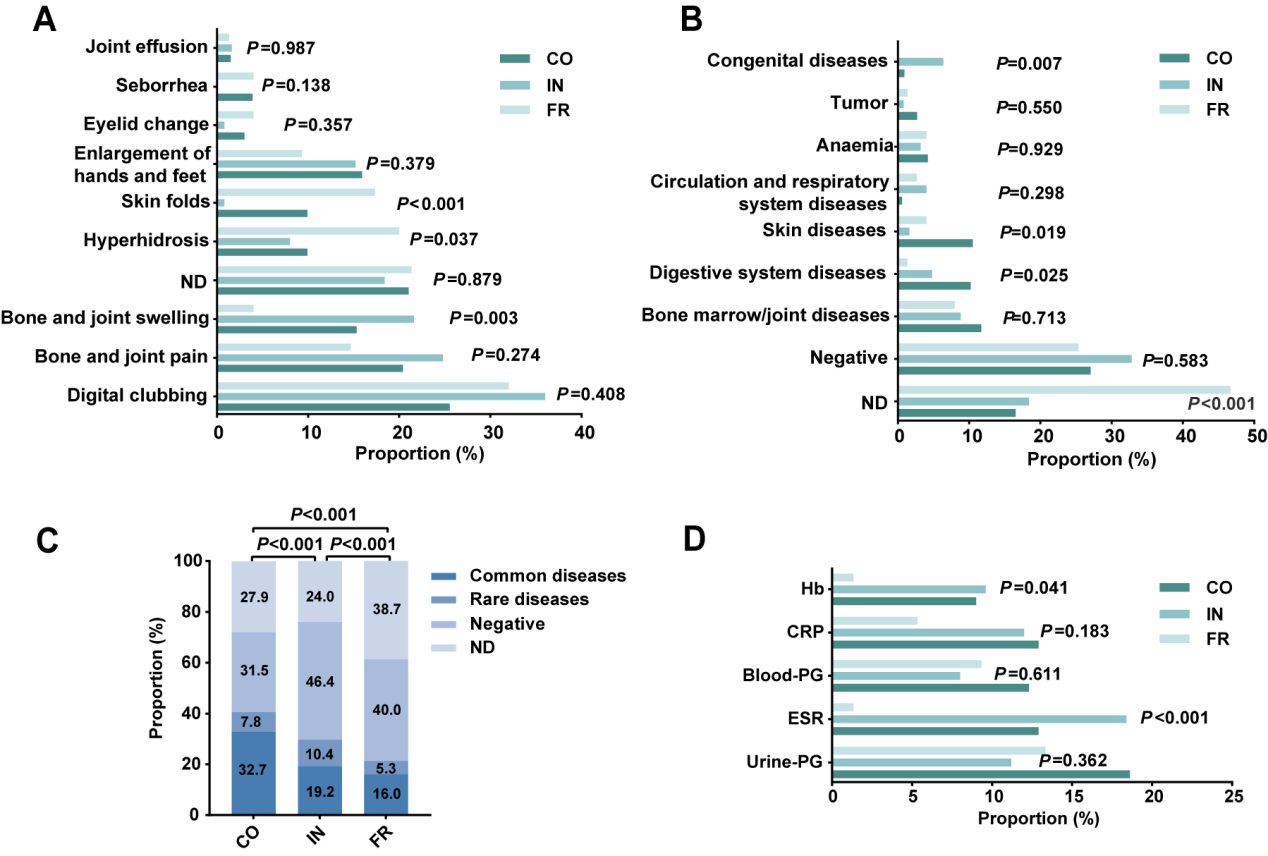

Supplement: Supplementary file 1 — Supplementary Material 1 [file 13023_2025_3722_MOESM1_ESM.docx]
